# Detection of the Linac Jaw face angle misalignment using high energy electron beam symmetry

**DOI:** 10.1002/acm2.70419

**Published:** 2025-12-10

**Authors:** Song Gao, Andres Cibrian, Jared Ohrt, Peter Balter

**Affiliations:** ^1^ Department of Radiation Physics The University of Texas MD Anderson Cancer Center Houston Texas USA

**Keywords:** acceptance and commissioning, beam symmetry, jaw face angle, linear accelerator

## Abstract

**Purpose:**

To demonstrate the usage of a high energy electron beam symmetry measured at different collimator angles to detect jaws face angle misalignments on Varian TrueBeam linear accelerators (linac) during acceptance and commissioning processes.

**Methods:**

During the linac acceptance, all beams were steered to a symmetric shape with the gantry and collimator at 0°. We noted that the symmetries of higher energies electrons (e.g., 16 MeV, 20 MeV) changed over 2% when measured with the collimator at 90° and/or 270° for two linacs commissioned within 5‐months of each other. Misaligned applicators were ruled out as the cause of this issue. Eventually, this was traced to the X‐jaw face angle not matching beam divergence. After the problem was identified and the jaws were realigned by manufacture's engineers and all beams were re‐steered to a symmetric shape at collimator 0° and verified at collimator 90° and 270°.

**Results:**

Prior to the manufacturer adjusting the jaw face angles, the maximum observed electron beam symmetry changes with 90° collimator rotation were 2.0% and 2.6% for linac 1 and linac 2, respectively. After realigning, the jaw face angles the maximum observed electron beam symmetry changes with 90° collimator rotation were within 1.0% for all beams on both linacs.

**Conclusion:**

We have demonstrated that changes in symmetry of high energy electron beam with 90° collimator rotation can signal that the jaw face angle is not properly aligned with beam divergence.

## 1 INTRODUCTION

After installation of the new linear accelerator (linac), there are two phases to making a linac ready for clinical usage: acceptance testing with tools, methods, and tolerances defined by the vendor, and commissioning by a qualified medical physicist with independent equipment. In the past, it was advised to include institution specific acceptance tests^1^, ^2^ into the purchase agreement, but this is very rare on modern machines. Vendors have also moved away from some tradition testing methods like film and water scans to more efficient and automated systems such as machine performance checks (MPC) and detector arrays. MPC is a manufacturer‐specific automated quality assurance system for checking machine performance. This accelerates the installation process but also places greater responsibility on the local physics staff to ensure that quality is not compromised by these newer methods. Any issues not identified during the vendor's acceptance testing ideally would be discovered during commissioning and these must be resolved before patient treatments begin often requiring the vendor to come back to the site delaying patient care.

In this work, we identify an issue with the secondary collimation that originated at the machine assembly. This issue was found on two separate Varian TrueBeam machines. We discuss why the issue was not identified in acceptance testing and how it was documented, reported to the vendor, and was eventually resolved.

**FIGURE 1:**
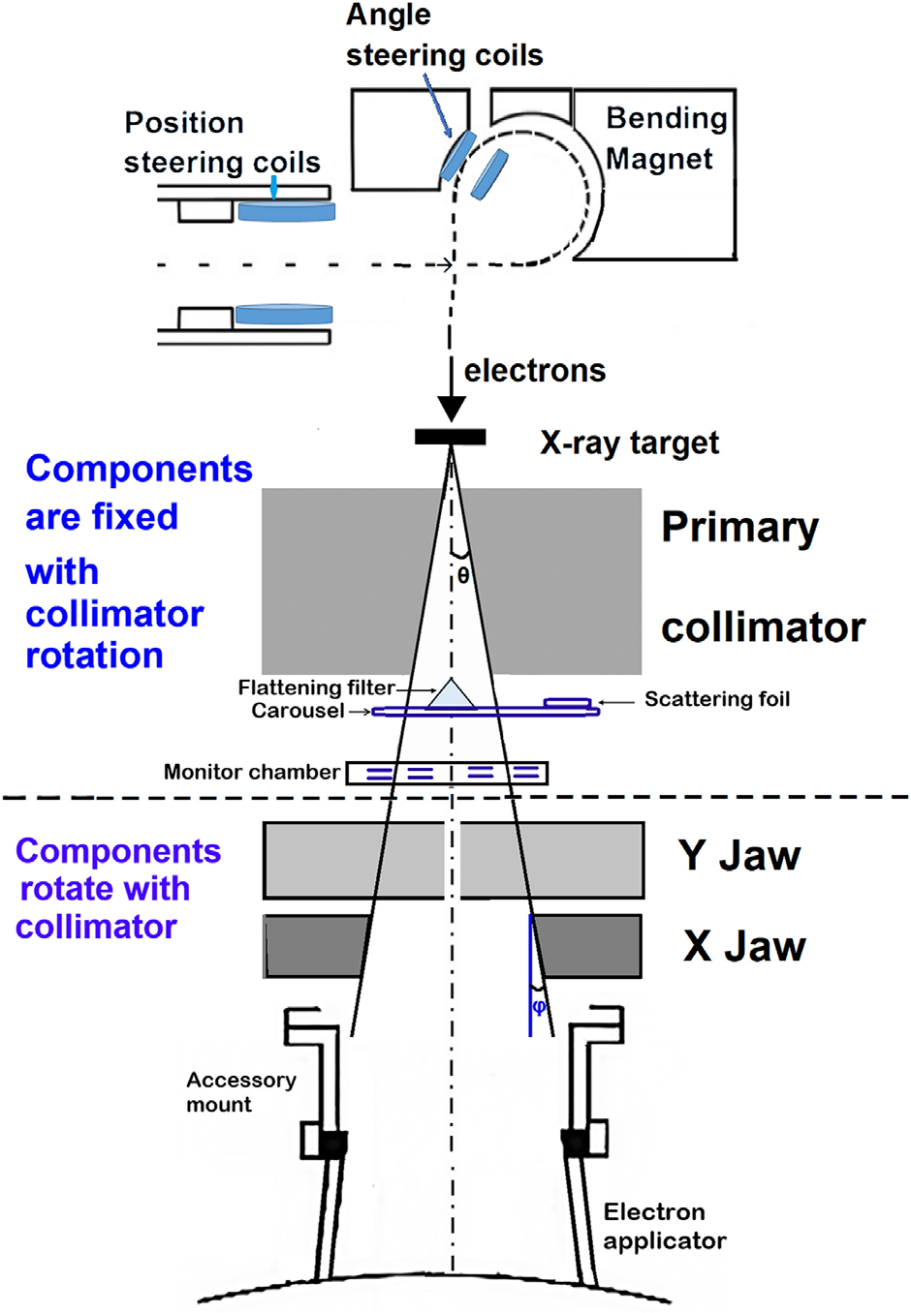
Schematic illustration of the Linac head geometry with the machine in photon mode, for electrons the photon target moves out of the beam and the flattening filter is replaced by duel scattering foils. Solid lines: the beam edge divergence, dot–dashed line: the beam central axis. The beam edge divergence angle: θ. The jaw face angle: ϕ. Current figure shows the orientation with the collimator at either 90° or 270° (X‐jaws are in‐plane with respect the beam creation axis). The physical thickness is 7.8 cm for both the X and Y jaws, and the distance from the X‐jaw midplane to the isocenter is 59.4 cm.

The secondary collimation system in a linac, namely collimators or jaws, consists of two pairs of metal blocks usually made from tungsten or lead alloy. When the jaws are extended, they tilt so that the jaw face angle equals the beam edge divergence angle (Figure 1). This ensures that the jaw face is parallel to the edge of the x‐ray beam.^3^, ^4^, ^5^ On the TrueBeam, the upper (Y) jaws move along a circular arc to keep the jaw face parallel to the beam divergence. The lower (X) jaws move along a linear trajectory and the jaw tilts to keep the front face parallel to beam divergence. If the face is not tilted properly to match the divergence angle, this would lead to changes in the beam characteristics due to the partial transmission through the jaw (photons) and differential scatter (electrons). Vendor acceptance testing for the jaws includes verifying multiple jaw positions versus the light field with the collimator at 0°. In addition, the coincidence of the light field and radiation field is checked using film. These tests only check the absolute position and parallelism of jaws, the jaw face angles are not explicitly tested but potentially could be inferred from other tests such as photon penumbra when using jaw collimation.

Beam steering is done to achieve symmetric beams that are aligned with the mechanical axis of the machine. On the TrueBeam the steering components (solenoids, bending magnet, target, primary collimator, flattening filter or scattering foils, and monitor ion chamber) are fixed with respect to collimator rotation and the position of the primary beam does not change with collimator angle. However, beam characteristics are affected by the secondary jaws and electron applicators (for electron beams) which rotate with the collimator (Figure 1). When steering is done it may include offsets away from the ideal mechanical axis to compensate for asymmetries in the beam profile caused by asymmetries in the jaws or the applicators. During acceptance testing, only a single collimator angle is used for both steering and testing thus asymmetries that become apparent when rotating the collimator are missed. Issues with asymmetric (bent) applicators are well known, but we have discovered that a misaligned jaw face angle can introduce similar effects resulting in electron beam symmetry changing significantly with collimator rotation. These subtle jaw face angle misalignments are not evident on other tests used for machine acceptance.

This study aims to evaluate the relationship between collimator rotation and electron beam symmetry as an indicator of jaw face misalignment. Two Varian TrueBeam linacs were found to have asymmetrical profiles of the high energy electron beams (16 and 20 MeV) with collimator rotation that was traced back to the X1 and X2 Jaw facings not being properly aligned with the beam edge divergence. Based on this experience, we propose that measuring changes in high energy electron beam symmetry with collimator rotation should be done to detect jaw face angles misalignment during linac acceptance and commissioning.

## 2 MATERIALS AND METHODS

### 2.1 Vendor acceptance testing

During the acceptance process of new linear accelerators beam steering is done by the vendor with their designated equipment. For the past several years, both Varian and Elekta have used an ionization chamber array (IC Profiler, Sun Nuclear, Melbourne, FL). For the acceptance testing on the Varian TrueBeam, all energies are evaluated with the gantry and collimator at 0° and are demonstrated to be within the vendor's specifications for flatness, symmetry, field‐size, penumbra, and energy (measured with a Quad Wedge Accessory),^6^ etc. We extended the acceptance testing to include verification of percent depth dose (PDD) and beam profiles using a 3D water scanning system, keeping gantry and collimator at 0°. The PDDs and the flatness and symmetry of the scanned profiles were found to be within the vendor's specifications and agreed with our institutional standards determined from previously commissioned linacs. However, when we did additional checks beyond acceptance testing with the collimator at 90° and/or 270° we found out that the symmetries for the 16 and 20 MeV electron beams were out of the 2% tolerance level recommended by TG‐142.^7^

### 2.2 Electron beam symmetry versus collimator angle

We measured the symmetry with an accurately calibrated^8^ IC Profiler (ICP) and verified the measurements with another accurately calibrated ICP. Statistical uncertainty in symmetry measurement with a well‐calibrated ICP was estimated to be ± 0.2% based on three repeated electron beam measurements. We measured the beam profiles using IC profiler with gantry at 0°, and collimator at 0°, 90°, and 270°. Profile data was acquired at SSD = 100 cm, 30 × 30 cm^2^ field, and 2 cm solid water buildup for photons, and a 25 × 25 cm^2^ applicator without buildup for electrons. All measurements presented in this work were acquired with the gantry at 0°. Checking the stability of beam profile with gantry angle for each beam is part of routine commissioning and annual QA. Since the beam symmetry may deviate as the linac's beam steering system rotates relative to the Earth's magnetic field, and movable components within the linac head may experience mechanical backlash at different gantry angles. The central axis (CAX) point‐to‐point difference symmetry metric was used for beam symmetry calculations,^8^ the symmetry (Sym) is defined as the maximum relative difference in intensity between pairs of mirror points across the CAX, normalized to the CAX intensity:

(1) $$Sym=\frac{D_{i}-D_{-i}}{D_{CAX}}\times 100\%$$

where $D_{i}$ is intensity at position “*i*”, $D_{-i}$ is intensity at opposing mirror point of “*i*” with respect to the CAX within 80% of the field size, $D_{CAX}$ is the intensity at CAX. Measuring at different collimator angles was made a standard procedure at our institution after issues were discovered with misaligned applicators on earlier commissioned machines. A new issue, unrelated to the applicators, was discovered using this method on two TrueBeam linacs installed over a 6‐month period at our institution, one with an HDMLC (Linac 1) and one with a millennium MLC (Linac 2). After initial beam steering by the vendor with the collimator at 0°, the symmetries for all photons and electron beams on both Linac 1 and Linac 2 were within ± 0.6%. When the collimator was rotated to 90°, the symmetries for lower energy electron beams (≤ 12 MeV) stayed within ± 1.0% (Linac 1) and ± 1.3% (Linac 2). However, for the 16 and 20 MeV beams the symmetries were 1.6% and −2.6%, respectively, for Linac 1 and 1.6% and 2.0% respectively for Linac 2 with the greatest discrepancy being along the measurement axis that was collimated by the X‐jaws, detailed results are in Table 1 (Section 3.1).

**TABLE 1:** 20 MeV beam flatness and symmetry for difference collimator angles before X jaws face angle adjustment. SSD = 100 cm, gantry at 0°, and 25 × 25 cm^2^ applicator.

Linac	Collimator angle (0°)	Crossplane			Inplane		
		Jaw	Flatness (%)	Symmetry (%)	Jaw	Flatness (%)	Symmetry (%)
Linac 1	0	X	0.9	−0.6	Y	1.4	−0.6
	90	Y	1.3	−0.1	X	1.5	−2.6
	270	Y	1.6	1.0	X	1.0	−0.4
Linac 2	0	X	1.2	−0.7	Y	1.3	0.5
	90	Y	2.1	−2.7	X	1.6	2.0
	270	Y	2.1	−2.6	X	1.4	−2.1

### 2.3 Working with vendor to identify the issue

Working with the vendor we additionally verified that the jaw positions were correct and that the electron applicators were straight and square. After eliminating these potential sources of this asymmetric profile error, the profile data was sent to the vendor's engineering specialists who indicated the problem could be caused by the misalignment of the jaw face angle.

The manufacture's engineers brought in a test jig to physically measure the jaw face angles and confirmed that both X1 and X2 jaw face angles were not aligned properly with the beam divergence. Both X1 and X2 jaw face angles were re‐aligned to meet the manufacturer's specification. Both the physical measurements using the test jig and the subsequent adjustment of the jaw face angles can only be performed by a manufacturer's technician. The jig is not generally available for purchase. Jaw face angle misalignments can occur in both the X and Y jaws; however, in the two cases examined in this study, the misalignments were limited to the X‐jaws. Because the Y‐jaws travel along an arc trajectory, they may be less prone to misalignment. After jaw face angles adjustments, all beams were re‐steered to point‐to‐point symmetries within ± 0.5% with the gantry and collimator at 0°. The profiles with collimator at 90° and 270° were measured for all photon and electron beams and the symmetries were all within ± 1.0% (Table 2).

**TABLE 2:** 20 MeV beam Flatness and symmetry for difference collimator angles after adjustment of the X jaws face angles. SSD = 100 cm, gantry at 0°, and 25 × 25 cm^2^ applicator.

Linac	Collimator angle (°)	X‐Axis			Y‐Axis		
		Jaw	Flatness (%)	Symmetry (%)	Jaw	Flatness (%)	Symmetry (%)
Linac 1	0	X	1.1	−0.1	Y	1.2	0.1
	90	Y	1.4	−0.5	X	1.3	−0.9
	270	Y	1.4	0.6	X	1.3	−0.7
Linac 2	0	X	1.2	0.3	Y	1.2	−0.5
	90	Y	1.2	0.0	X	1.2	−0.3
	270	Y	1.2	−0.1	X	1.3	−0.9

## 3 RESULTS AND DISCUSSION

### 3.1 Electron beam symmetry versus collimator angle at machine acceptance

**FIGURE 2:**
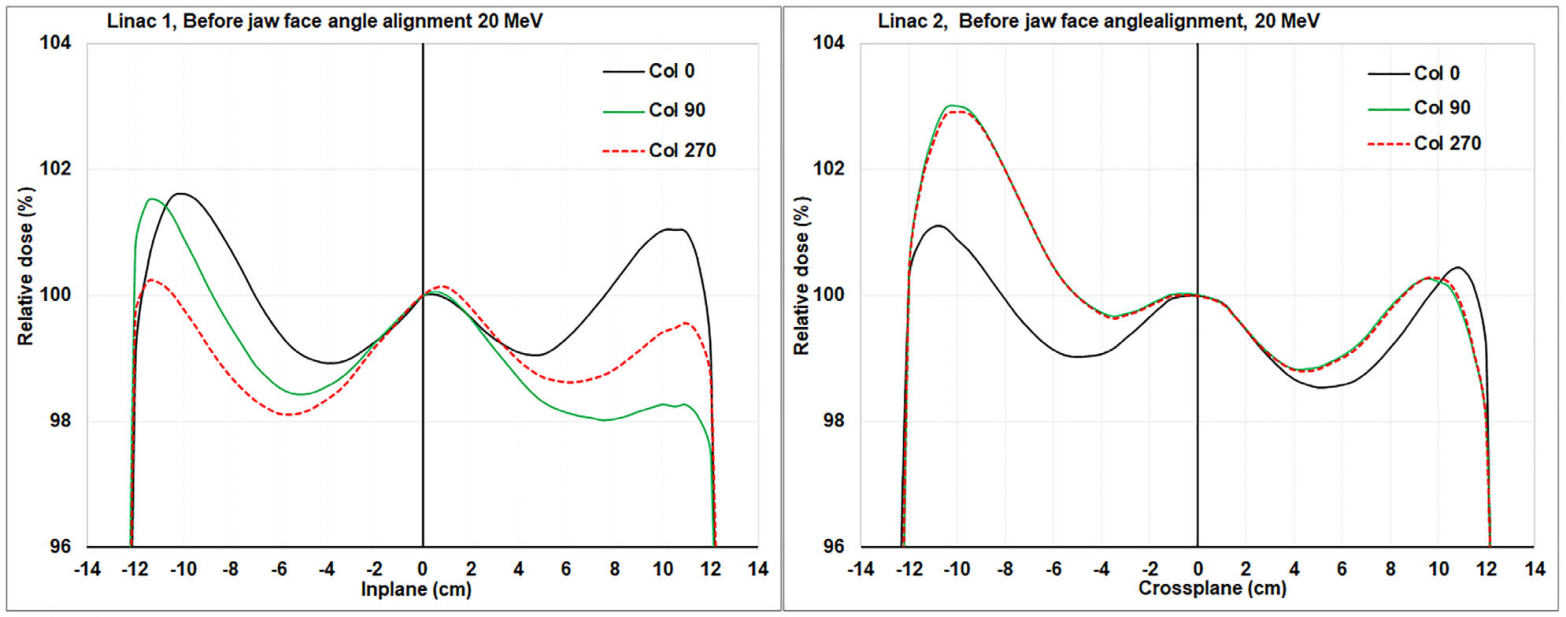
The 20 MeV beam profiles (enlarged) at different collimator angles (0°, 90°, and 270°) before X jaw face angles adjustments for Linac 1 (Inplane) and Linac 2 (Crossplane).

The symmetries for all photons and electrons were steered to be within ± 0.6% with the collimator at 0°. For photons, when the collimator was rotated to 90° and 270° the symmetries were within ± 0.9%, and the symmetry differences between those measurements with collimator at 0° and collimator at 90°/270° were within 0.5% for both linacs. For low energy beams (≤ 12 MeV) when the collimator was rotated to 90° and 270° the symmetries were within ± 1.0% (Linac 1) and 1.3% (Linac 2), and the symmetry differences between those measurements with collimator at 0° and collimator at 90°/270° were within 0.7% (Linac 1) and 0.9% (Linac 2). For higher energy electron beam (16 and 20 MeV) when the collimator was rotated to 90° and 270° the symmetries and beam profiles showed larger changes with the 20 Mev showing the largest effect (Table 1 and Figure 2). When reviewing the cross‐plane symmetry data, it becomes clear that the beam was steered at a 0° collimator angle, with the X‐jaws providing collimation and scatter. However, an incorrect X‐jaw face angle caused the beam to be steered improperly, as it had been offset to compensate for this face‐angle issue. This is demonstrated by the maximum change in symmetry of 1.6% and 2.0% for linac 1 and linac 2 when the collimator is rotated 90° (to 90° or 270°) and the Y‐jaws become the ones affecting collimation and scatter in this measurement plane. Conversely, the in‐plane steering was done based on the Y‐jaws which did not have the face‐angle issue and the raw beam was in the correct location but when the collimator is rotated to put the X‐jaws in this plane the symmetry changes by a maximum of 2.0% and 2.6% for linac 1 and linac 2 due to improper face angle on the X‐jaws causing asymmetrical scatter. The symmetries and beam profiles exhibited more significant changes when the collimator was rotated to 90° and 270°. This suggests a misalignment in the jaw face angles. Both Linac 1 and Linac 2 have incorrect jaw face angles, though they are tilted differently, resulting in distinct beam profile behaviors at the 90° and 270° collimator angles.

### 3.2 Electron beam symmetry versus collimator angle after jaw face angle re‐alignment

**FIGURE 3:**
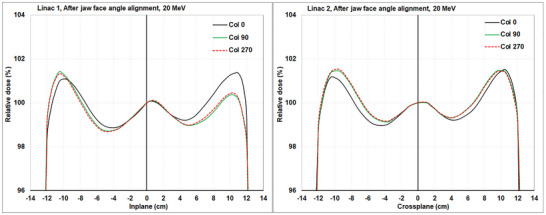
The 20 MeV beam profiles (enlarged) at different collimator angles (0°, 90°, and 270°) after X jaw face angles adjustments for Linac 1 (Inplane) and Linac 2 (Crossplane).

After both X1 and X2 jaw face angles had been adjusted to meet the manufacture's specification the beams were steered with the gantry and collimator at 0° to achieve symmetry within ± 0.5%. The differences in symmetry between the profiles measured with collimator at 0° and collimator at 90°/270° were now within 1.0% for all beams with the largest disagreements/profiles change still occurring for the 20 MeV beam (Table 2 and Figure 3).

### 3.3 Discussion

Electron beam symmetry can change with collimator angle due to two factors beyond initial steering and jaw position calibration: the electron applicator being asymmetric with respect to the axis of collimator rotation and the jaw face angle not matching beam divergence.

The effect of electron applicator asymmetry^9^ is most pronounced at lower energies that have increased scattering close to the measurement surface. This behavior is well known and may vary over the lifetime of the machine because of normal use and handling of the applicators. Mechanical checks performed by physicists or engineers can identify these issues.

This work demonstrates that the jaw face angle not matching beam divergence can also cause asymmetries in electron beams with collimator rotation. We found this is most pronounced at higher energies (16 and 20 MeV). This is not a commonly observed phenomenon and results from initial machine setup at the factory and is not likely to change during the lifetime of the machine outside of major service.

A previous study has demonstrated the effect of electrons scattered off collimators on beam profiles.^5^ They noted that the energy of the scattered electrons is roughly 40% of the primary beam's mean energy, which may explain why this effect was not seen at the lower energies used in the present work. At those energies, the scattered electrons would have been stopped before reaching the detectors in the ICP, which sit at a water‐equivalent depth of 0.9 cm. Another reason this effect may be smaller from lower energies is that the scatter off the jaws for the lower energies is less forward peaked and may not reach the plane of the measurement. A third explanation for the lower sensitivity at lower energies is the position of the jaws. On the TrueBeam for the 25 × 25 cm^2^ applicator the low energy electrons have jaws settings much larger than the nominal field size (6 MeV jaws are set to 32 × 32 cm^2^) while for high energy electron the jaw settings (20 MeV jaws are set to 27 × 27 cm^2^) are closer to the measured field size (25 × 25 cm^2^). Due to this, scatter from the jaws is more likely to impact the measured profiles so the contributions of electrons scattering from the jaw faces can cause a more pronounced asymmetry.

In summary, changes in the symmetry of high energy electron beams with different collimator angles primarily arise from the interaction of electron scattering with the specific design and geometry of the collimation system. The electron beam symmetry information can be used to ensure the geometry of the collimation system are properly aligned for achieving the desired beam characteristics. The practical benefit of this method lies in its ability to diagnose jaw face angle misalignment in a fast and non‐invasive manner. The significant advantages of identifying such errors during acceptance testing include preventing systematic errors, significantly reducing machine downtime, shortening commissioning time, clarifying responsibility to ensure vendors address performance issues, and establishing a correct baseline. We acknowledge that the limitation of this study lies in the fact that it is based on data from two linacs of the same design at a single institution measured with a single type of detector array.

## 4 CONCLUSIONS

The manufacturer's tests for jaw face angle misalignment are highly invasive, require machine down time, and are not part of routine acceptance testing and commissioning. We have demonstrated that the improperly set jaw face angles can be identified based upon the change in symmetries of high energy electron beams with collimator angle. We recommend checking the consistency of high‐energy electron beam symmetries at different collimator angles during linac acceptance, commissioning, and the annual QA process.

## AUTHOR CONTRIBUTIONS

Song Gao designed the study, collected measurement data, analyzed results, wrote and edited the manuscript. Andres Cibrian supported to identify and resolve the problem, edited the manuscript. Jared Ohrt wrote and edited the manuscript. Peter Balter designed the study, wrote and edited the manuscript.

## CONFLICT OF INTEREST STATEMENT

None.

## Data Availability

The data that support the findings of this study are available from the corresponding author upon reasonable request.
